# The New Insurance Books

**Published:** 1914-08-01

**Authors:** 


### NATIONAL INSURANCE ACT DEVELOPMENTS.

### The New Insurance Books.

When the National Insurance Act came into operation in July 1912, one of the first duties that devolved upon the approved societies was to furnish their members with "Insurance books." The primary object of these Insurance books was to provide a record of the contributions paid and the benefits received by the insured persons, very much in the same way as the bank books issued to depositors in the Post Office Savings Bank act as a record of amounts deposited and withdrawn from time to time. The books were, however, designed with the important secondary object of furnishing information, in a compact form, of the rights and duties appertaining to the insured persons by virtue of their insurance. In general, an Insurance book was to remain in the possession of the contributor to whom it belonged, but for the purpose of making the necessary entries it was to be deposited with his society from time to time as might be required.

The first Insurance books were originally designed to cover the first five quarters of the operation of the Act—that is to say, until October 1913; but, owing to the alterations imposed by the Act of 1913, it was found necessary to employ them, as an expedient, for a further quarter, thus bringing the record of contributions paid up to January 11 of the present year.

### The General Character of the New Books.

New Insurance books are now in course of issue, and one should be in the hands of every insured person within the next few weeks. The books are designed with the same objects as the original, but in form they are essentially different. In one respect—namely, that of size—the new books are doubtless an improvement upon the old, as the size has been substantially reduced. This reduction in length and breadth may not at first sight appear to be of any consequence, but beyond the saving in material—card and paper—which goes to make up the books—which saving, in total, is likely to be very considerable—there is the important advantage that it is probable that less difficulty will be experienced in finding envelopes to take the books when it is necessary to send them through the post. It is curious that such a matter as the want of an envelope of appropriate size should influence the expeditious transaction of business, but it is nevertheless a fact that many insured persons have delayed the dispatch of their Insurance books to their societies for this reason, and have suffered delay in the settlement of their claims in consequence.

But although the size of the new books is in their favour, it must be admitted that the contents are so complicated as to make it practically certain that only a very small proportion of the insured population will take the trouble to read the regulations that are set forth therein. This is a serious disadvantage, because it implies that the vast majority of the insured persons will be unfamiliar with their rights and duties. In its turn this will mean disappointment or dissatisfaction when the insured persons find that by not complying with the regulations they entirely lose, or suffer a reduction in, the benefits to which they would have become entitled if they had had full knowledge of the regulations, and had acted accordingly. More than this, it is clear that the complication in administrative machinery is sure to lead to extensive correspondence between the members and their societies, and with this the expenses of administration will be increased, so that the burden on the societies under this head will be enhanced.

### Personal Explanations.

It seems to have been forgotten that in order that the books should reach the maximum of utility adequacy of explanation should have been sufficiently tempered by simplicity of form, so that no unnecessary deterrent should have been imposed on the reading of the instructions. There is, however, one feature of the administration of the Act that may mitigate to some extent the disadvantage of the complexity of the new Insurance books—namely, the fact that a large proportion of the insured persons are in direct touch with local representatives of their societies. We know, as a matter of fact, that several of the largest societies have given explicit instructions to their representatives to bring to the notice of the members, with whom they are individually in contact, the more important provisions of the new regulations contained in the Insurance books, and to point out to them the regulations affecting their rights to benefit. In this way the personal visit of the agent of the approved society will assist in preventing non-compliance with the rules arising either through ignorance or neglect.

### The Reason for the New Form of Insurance Book.

The principal reason for the altered form of the Insurance books is to give effect to the new regulations that have quite recently been adopted by the Insurance Commissioners with regard to arrears of contribution. Indeed, the greatest prominence is given in the books to the payment of arrears and the effect of arrears on benefits, and it is, doubtless, because these new regulations are themselves so complex that the Insurance books reflect the complexity.

It will be remembered that the scheme for giving effect to arrears of contribution set forth in the principal Act made it necessary to calculate the average arrears, and the reductions in the rates of benefit were made to depend on the average so calculated. So strong was the protest against the impracticability of the scheme that it never came into operation, and the new scheme has been devised as an efficient and, withal, as a more simple plan for giving effect to arrears. Accepting the new scheme as a simplification of the original plan, one is tempted to wonder how complex the original scheme would have been if it had actually come into operation.

### The Calculation and Payment of Arrears.

The process adopted under the new scheme is briefly as follows. The period from July 1 in one year to June 30 in the year following is to be known as the "Contribution Year." At the end of each contribution year, or as soon after as possible, the Insurance books are to be made up by the entry therein of the number of contributions paid, the number of contributions not payable on account of sickness, and the number of arrears paid, if any. If the total of these three items falls short of the total number of weeks in the contribution year the difference is the "Gross arrears" due by the member at the end of the contribution year.

The gross arrears are, however, to be modified by reference to the number of "Reserve contributions" standing to the credit of the member. These reserve contributions depend upon the date of the member's entry into insurance. Thus for those who entered when the principal Act came into operation the reserve contributions credited at the outset will be six, while for those who entered during the second half of the last contribution year the credit will be one only.

Having deducted the reserve contributions from the gross arrears the difference, if any, will be the amount of the "Penalty arrears" at the close of the contribution year. If the reserve contributions are greater than the gross arrears the difference will be the amount of the "Reserve balance" which is to be carried forward in the member's favour to be set against any arrears that may accrue in future years.

At the end of each contribution year the approved societies are allowed a period of six weeks in which to make up the accounts of their members, and before the expiration of that period they are required to return the Insurance books to the members concerned, and to notify any who may have penalty arrears debited to them as to the effect of those arrears on their right to benefit unless they are paid or reduced before the expiration of the "Period of grace," which is the thirteen weeks following the end of the contribution year. At the same time as the notification is sent an arrears card must also be furnished, so that the member may be in a position by affixing the necessary Health Insurance stamps to pay the arrears or some part thereof within the period of grace should he so desire.

### The Effect of Arrears on Benefits.

At the end of the period of grace, which will expire this year on October 4, the account will be finally made up, and the Insurance book belonging to each insured person will show as on that date the amount of his "Net penalty arrears" or the "Reserve balance" carried forward.

Effect is then to be given to the net penalty arrears in the "Penalty year" beginning on, or immediately after, November 1 immediately following. Penalty arrears, unlike the reserve balance of contributions, are not carried forward from one year to the next, but are wiped out by the reduction or suspension of benefit to which the member is liable in the following penalty year.

### Suspension of Benefit.

The reduction or suspension of benefit depends in amount on the actual number of the net penalty arrears debited against the member. Thus if net penalty arrears do not exceed sixteen the rate of sickness or disablement benefit for the first six weeks for which the benefit is payable in the penalty year will be reduced according to the printed scale that is set forth in the insurance book. For example, if a man who would ordinarily be entitled to sickness benefit at the rate of 10s. a week has twelve penalty arrears against him, he will, if he should claim benefit during the following penalty year, be entitled for the first six weeks of his claim to 3s. a week only. The full rate of benefit will be payable after the first six weeks of benefit, and no reduction will be made in other benefits.

As the number of net penalty arrears increases, so also does the severity of the penalty. Thus when the penalty arrears exceed sixteen, but do not exceed twenty, sickness and disablement benefits will be entirely suspended for the first six weeks, for which the benefit would otherwise be payable in the following penalty year. And if the penalty arrears exceed twenty, but do not exceed twenty-six, these two benefits will be entirely suspended throughout the whole of the penalty year. In neither of these cases, however, are the other benefits affected. But if the penalty arrears exceed twenty-six, then all benefits, including medical and sanatorium benefits, will be suspended for the whole penalty year.

### Is a Simpler Scheme Feasible?

When the general outline of the system has been grasped there is very little difficulty in understanding the operation of the regulations for the reduction or suspension of benefits. It must be admitted, however, that the system leaves much to be desired in the matter of simplicity, and simplicity in detail is an essential quality for the permanent success of any general scheme of insurance. This has been proved in the organisation of the old Friendly Societies and the Industrial Assurance companies. It has always been, and still is, the practice to consider a member of a Friendly Society, or an assured person, either as in full benefit or out of benefit. Before the assurance can lapse due notification has to be given of the impending loss of benefit unless arrears are paid before a stipulated date, and instructions are furnished as to the requirements for reinstatement if the arrears are not paid in time. It is true that the assurances referred to are of a voluntary character, but it may be asked if the national compulsory insurance is so far different from the voluntary systems as to require such an essentially different plan for the treatment of arrears.

